# What could be the potential reasons for relatively low coronavirus disease 2019 (COVID-19) fatality rates in Africa? The case for Ethiopia

**DOI:** 10.7189/jogh.11.03057

**Published:** 2021-04-17

**Authors:** Taddese Alemu Zerfu, Amare Abera Tareke

**Affiliations:** 1 International livestock research institute (ILRI), Nairobi, Kenya; 2 Global Academy of Agriculture & Food Security (GAAFS), University of Edinburgh, UK; 3 Physiology unit, Department of Biomedical Sciences, College of Medicine and Health Sciences, Wollo University, Dessie, Ethiopia

In December 2019, an outbreak of severe acute respiratory syndrome coronavirus 2 (SARS-Cov-2) infection occurred in China that later spread across the globe. On February 12, 2020, World Health Organization (WHO) officially named the disease caused by the novel coronavirus as coronavirus Disease 2019 (COVID-19) [1].

Coronaviruses (CoVs), which are RNA viruses, generally cause enteric and respiratory diseases in humans [2]. Despite most human CoVs cause mild respiratory diseases, the worldwide spread of two previously unrecognized CoVs, the severe acute respiratory syndrome CoV (SARS-CoV) and Middle East respiratory syndrome CoV (MERS-CoV) have called global attention to the lethal potential of human CoVs [3].

**Figure:**
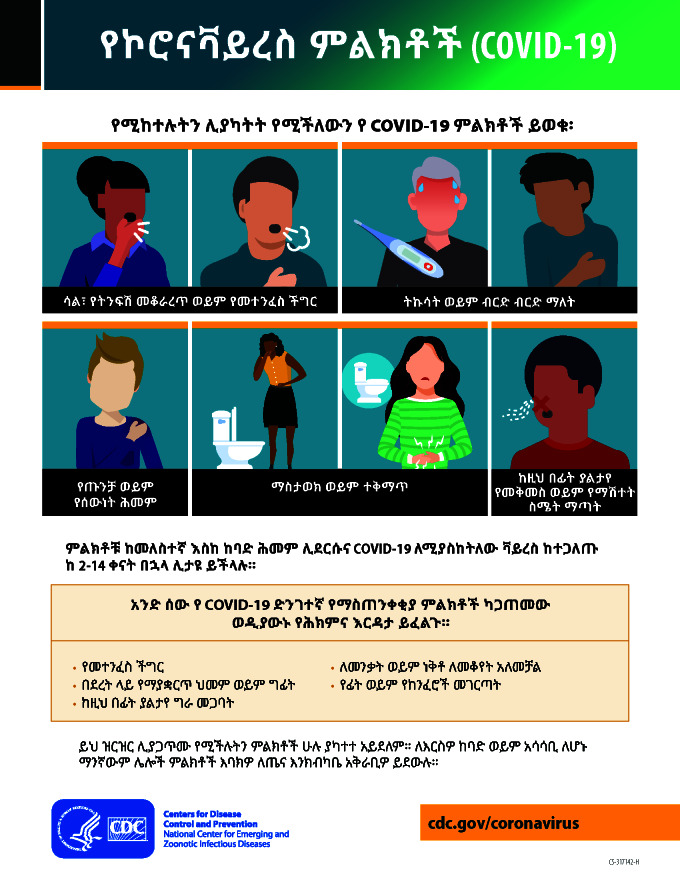
Photo: Symptoms of COVID-19 (Amharic). Source: National Center for Immunization and Respiratory Diseases (NCIRD), Division of Viral Diseases, via Wikimedia Commons.

The COVID-19 pandemic has now involved numerous low-to-middle-income countries (LMICs). The health care systems in LMICs face serious constraints in capacity and accessibility during normal times. The expectation was, this would be aggravated during an outbreak, leading to worse clinical outcomes. Moreover; 69% of the global population aged 60 and above live in LMICs. These older persons are at increased risk of severe COVID-19 and mortality [4].

LMICs including Ethiopia, lack time and finance for swift uptake of new technologies in a more urgent and pragmatic manner. A limited number of isolation beds, accredited test laboratories/sites, especially in suburbs and regional hospitals, lack of point-of-care-certified test kits at the frontlines and community, insufficient ICU ventilators, insufficient oxygen supply, insufficient medications are bottlenecks in overcoming COVID 19. These factors and the experience in developed countries pushed the Ethiopian national estimate of mortality to be high.

Since the onset of the disease besides the number of people infected, the fatality rate is variable across countries. Previously, global mortality rates over time using a 14-day delay estimate were 5.7%, other literature also found the mortality rate to be as high as 20% in areas like Wuhan the epicenter of the outbreak [5,6]. Currently, COVID 19 mortality rate has passed 10% in some countries, whilst in Ethiopia until October 19, 2020, is 1.52% [7,8] (**Figure 1**). Here we present the most probable reasons for the relatively low mortality rate in Ethiopia.

**Figure 1:**
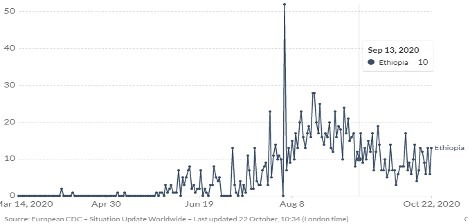
Trends in COVID-19 related deaths in Ethiopia, March-October 2020. The figure depicts rise in COVID-19 mortality rate from March to August, and fall from August to October.

## REPEATED EXPOSURE TO SIMILAR RESPIRATORY INFECTIONS

In Ethiopia, many people have had repeated episodes of similar respiratory tract infections, long before the appearance of COVID 19, in the form of common cold and influenza [9]. Evidence shows that presence of memory B cells (MBCs) that could recognize SARS-CoV-2 can rapidly produce antibodies [10]. According to the latest evidence, past infection with similar virus generates both IgG and IgG MBCs against the novel receptor-binding domain and the conserved S2 subunit of the SARS-CoV-2 spike protein. Thus, even if antibody levels wane, long-lived MBCs remain to mediate rapid antibody production. In populations like Ethiopia, where frequent influenza infection is very common, antibodies that target specific part of spike proteins which exist in corona viruses could be crucial for decreasing disease severity and mortality.

## POPULATION STRUCTURE AND ASSOCIATED BURDEN OF CHRONIC NON-COMMUNICABLE DISEASES

Several population characteristics are recognized as contributors for severity and mortality for COVID-19. Mounting evidence shows that a high burden of severe disease and death from COVID-19 has been consistently observed in older patients, especially those with pre-existing medical co-morbidities [11,12]. Ethiopia, like other sub-Saharan Africa countries, has a distinct population structure with a great majority of the population being below the age of 30. Only 3.52% of Ethiopian population was above 65 years in 2019. It is also a well-known fact that these chronic comorbid conditions, although emerging, are not highly prevalent in Ethiopia.

## RELATIVE LOW AIR POLLUTION IN ETHIOPIA

Air pollution in some countries might have played a significant role in COVID-19 related mortality. Long-term average exposure to fine particulate matter significantly increased COVID-19 deaths in the United States [13]. In China, a significant relationship between air pollution and COVID-19 infection was observed, which could partially explain the effect of national lockdown and provide implications for the control and prevention of this novel disease. Another example was, day-by-day changes of pollutant concentration were positively linked with the number of infected individuals in Italy; likewise, the number of confirmed cases was extremely higher in cities with more than 100 days of air pollution than cities with cleaner air [14]. Fortunately; Ethiopia is one of the countries with the lowest pollutant and hydrocarbon gas emissions contributing little to global warming and air pollution, making the country at lower risk of COVID-19 related to this.

## WEATHER AND ENVIRONMENTAL TEMPERATURE

Evidence has also supported low environmental temperature contributing to COVID 19 transmission [11,15]. To this end, Ethiopia found in sub-Saharan tropical Africa, has an average temperature greater than most countries with notably high fatality rate. Although, it is still debatable for corona virus, vitamin D deficiency in colder climates may trigger the disease in those regions [16].

## LOW SMOKING RATE

Clean lung has been identified as one of the best indicators for better prognosis after acquiring corona virus. Smoking is detrimental to the immune system and its responsiveness to infections, making smokers more vulnerable to infectious diseases [17]. Current evidence suggests that the severity of COVID-19 disease is higher among smokers [18]. Smoking impairs lung function, making it more difficult for the body to fight off respiratory disease due to the new coronavirus. A recent meta-analysis of evidence from seven studies found a statistically significant association between smoking and severity of COVID-19 outcomes amongst patients [18]. In Ethiopia, even though there is a growing number of smokers, about 8.9% of the total population are currently smoking, the burden and daily intake doses are quite below the global and African levels [19].

In conclusion, with little available evidence and test rates in the country, Ethiopia appears to be experiencing one of the lowest case-fatality rates attributed to corona virus. This could be explained by various individual, household, and community level factors. Critical assessment of such possible factors revealed that population’s previous repeated experience of exposure to similar respiratory infections, the structure of the national population (massively dominated by young population), level of air pollution in the country, the weather and environmental temperature conditions, and the level of smokers could be listed as key reasons to the observed relative low mortality levels. A single factor may not be sufficient to explain the relatively low mortality rate, but probably a combination of several of them. Some factors which hypothesized here, such as the possibility of prior antibody presence, requires verification on Ethiopian population.
